# A taste of wilderness: supplementary feeding of red deer (*Cervus elaphus*) increases individual bacterial microbiota diversity but lowers abundance of important gut symbionts

**DOI:** 10.1186/s42523-024-00315-6

**Published:** 2024-05-14

**Authors:** Luis Víquez-R, Maik Henrich, Vanessa Riegel, Marvin Bader, Kerstin Wilhelm, Marco Heurich, Simone Sommer

**Affiliations:** 1 Institute of Evolutionary Ecology and Conservation Genomics, Ulm University, Ulm, Baden-Württemberg Germany; 2 Department of Biology, Bucknell University, Lewisburg, PA USA; 3 Department of National Park Monitoring and Animal Management, Bavarian Forest National Park, Grafenau, Bayern Germany; 4 Chair of Wildlife Ecology and Wildlife Management, University of Freiburg, Freiburg, Baden-Württemberg Germany; 5 Albert-Ludwigs University, Freiburg, Baden-Württemberg Germany; 6 Institute for Forest and Wildlife Management, Inland Norway University of Applied Sciences, Koppang, NO-34 Norway

**Keywords:** 16S rRNA sequencing, Wildlife management, Winter enclosures, Diet supplementation, Artiodactyla, Bavarian national park/Germany, Prevotellaceae, *Roseburia*

## Abstract

The gut microbiome plays a crucial role in the health and well-being of animals. It is especially critical for ruminants that depend on this bacterial community for digesting their food. In this study, we investigated the effects of management conditions and supplemental feeding on the gut bacterial microbiota of red deer (*Cervus elaphus*) in the Bavarian Forest National Park, Germany. Fecal samples were collected from free-ranging deer, deer within winter enclosures, and deer in permanent enclosures. The samples were analyzed by high-throughput sequencing of the 16 S rRNA gene. The results showed that the gut bacterial microbiota differed in diversity, abundance, and heterogeneity within and between the various management groups. Free-ranging deer exhibited lower alpha diversity compared with deer in enclosures, probably because of the food supplementation available to the animals within the enclosures. Free-living individuals also showed the highest beta diversity, indicating greater variability in foraging grounds and plant species selection. Moreover, free-ranging deer had the lowest abundance of potentially pathogenic bacterial taxa, suggesting a healthier gut microbiome. Winter-gated deer, which spent some time in enclosures, exhibited intermediate characteristics between free-ranging and all-year-gated deer. These findings suggest that the winter enclosure management strategy, including supplementary feeding with processed plants and crops, has a significant impact on the gut microbiome composition of red deer. Overall, this study provides important insights into the effects of management conditions, particularly winter enclosure practices, on the gut microbiome of red deer. Understanding these effects is crucial for assessing the potential health implications of management strategies and highlights the value of microbiota investigations as health marker.

**Supplementary Information:**

The online version contains supplementary material available at 10.1186/s42523-024-00315-6.

## Introduction

In recent years, the holobiont concept has established the idea that gut microorganisms are fully integrated and essential fellows of their hosts [1, 2]. In some particular cases, this relationship is so intertwined that primary and essential functions for host survival are hard-wired not in their own DNA but in the DNA of their symbionts [3]. In ruminants, gut bacteria are the first to interact with forage ingested by the host animals [4]. In the foregut, a plethora of bacteria contributes to this interaction. Clostridiales help to break down proteins, pectins, and cellulose, whereas *Prevotella* degrades hemicellulose in the rumen [5]. Animals benefit from a healthy gut, but not all microorganisms are beneficial for the host. Inside the gut, the available space for anchoring to the mucosa is limited, and bacteria and other microorganisms are in a constant struggle, competing with newcomers in the search for a niche in which to settle [6].

This tug of war results in a chemical struggle between various strains of bacteria and can, in turn, have detrimental effects on the host by causing a dysbiosis, a shift in bacterial species community and abundance pattern beyond the normal range of variation [7–9]. The host has an interest in holding on to beneficial microorganisms, while repelling potential invaders and pathogens [10]. The immune system of the host curates the relationship between the host and its endosymbionts by surveillance of the intestinal lumen and the release of anti-microbial peptides and IgA antibodies [11]. A healthy and balanced gut microbiome therefore not only provides benefits in terms of nutrient availability, but also interacts with the host’s immune system [10, 12, 13].

For most mammals, the main route of exposure to new microorganisms and pathogens is through the food that they consume and, to a lesser extent, through their associations with other animals of the same or different species [14]. However, the diet of many wild free-ranging animals might deviate from the “natural” state to which they are evolutionarily adapted, because of anthropogenic influences [9, 15]. Such dietary changes might strongly impact the composition and diversity of the gut microbiome within individuals (alpha diversity), causing a potential decline in beneficial microbial functions [16, 17]. Furthermore, the diversity of the gut microbiome between individuals (beta diversity) can also be affected. On the one hand, stressors might reduce the ability of animals to counteract stochastic changes of the composition of the microbiome [17, 18]. On the other hand, individual differences in the selection of food sources between animals potentially contribute to a natural degree of beta diversity that may be reduced if most individuals in the population consumed the same anthropogenic items. A decrease in microbiome alpha diversity and a change in beta diversity might consequently be indicators of declining animal health across a population [17, 18].

Red deer (*Cervus elaphus*) are the second-most widespread wild ungulate species in Europe [19] and important ecosystem “engineers” that can promote biodiversity, e.g., by browsing dominant plant species, by creating forest openings, by contributing to the dispersal of plant seeds, and by providing food resources for large predators and scavengers [19–23]. However, many behaviors of red deer, such as browsing and bark stripping might be beneficial ecologically to a certain degree, but also lead to damages and economic losses for forestry and agriculture [19]. Red deer populations are therefore intensely managed, like other ungulate species in the northern hemisphere such as elk (*Cervus canadensis*), moose (*Alces alces*), roe deer (*Capreolus capreolus*), and fallow deer (*Dama dama*) [24]. In many areas, hunting is used as a method of population control [24]. The downstream effect of this practice is that animals become more reclusive. Such behavioral changes can substantially impact their diet, as the grasses, herbs, and shrubs from open foraging grounds are replaced by tree leaves, buds, and bark [25], and their exposure to new bacterial sources affects their gut microbiome composition. In addition, red deer management often involves the provisioning of supplementary food to decrease winter mortality and damage to forest regeneration, although evidence for the effectiveness of the latter is limited [24]. In mountainous areas, such feeding stations are often fenced to restrict the movement of the animals and to limit browsing damage in the majority of the area, forming so-called winter enclosures [26]. Although this management strategy also leads to extensive browsing in and around the enclosures [22], the composition of the animals’ winter diet will change drastically compared with that in a natural state, incorporating a high proportion of processed plants (hay or silage) and crops. The aggregation of animals at feeding sites may also favor the spread and persistence of diseases [27, 28].

In this study, we used a 16 S rRNA gene high-throughput sequencing approach to investigate the effects of a management strategy on the bacterial gut microbiota of red deer (*Cervus elaphus*) in the Bavarian Forest National Park (Germany). We compared animals living permanently in enclosures, animals staying over winter in enclosures, and free-living animals. Our specific aims were (i) to test whether the gut bacterial microbiota differed in diversity, abundance, and heterogeneity within and between treatment groups, (ii) to identify any individual bacterial taxa that were differentially abundant because of the different management conditions, and (iii) to compare those taxa with existing bacterial taxa present in databases and reported in the literature in order to reveal potential health implications. We hypothesized that a higher alpha diversity would characterize the gut microbiome of free-living deer compared with that of animals spending time in captivity, the supplementary feed being less diverse than the natural forage. Furthermore, we expected that the free-living deer would show the highest beta diversity, because the selection of foraging grounds and plant species often varies the most between individuals. Additionally, we predicted that the free-living deer would show the lowest abundance of potentially pathogenic bacterial taxa. The winter-gated individuals should take an interim position between free-ranging and all-year-gated animals. Our study provides important insights concerning the effect of the winter enclosure management practice in terms of diet supplementation on the microbiome of individuals and its meaning for population health.

## Methods

### Red deer management conditions and fecal sample collection

**Fig. 1:**
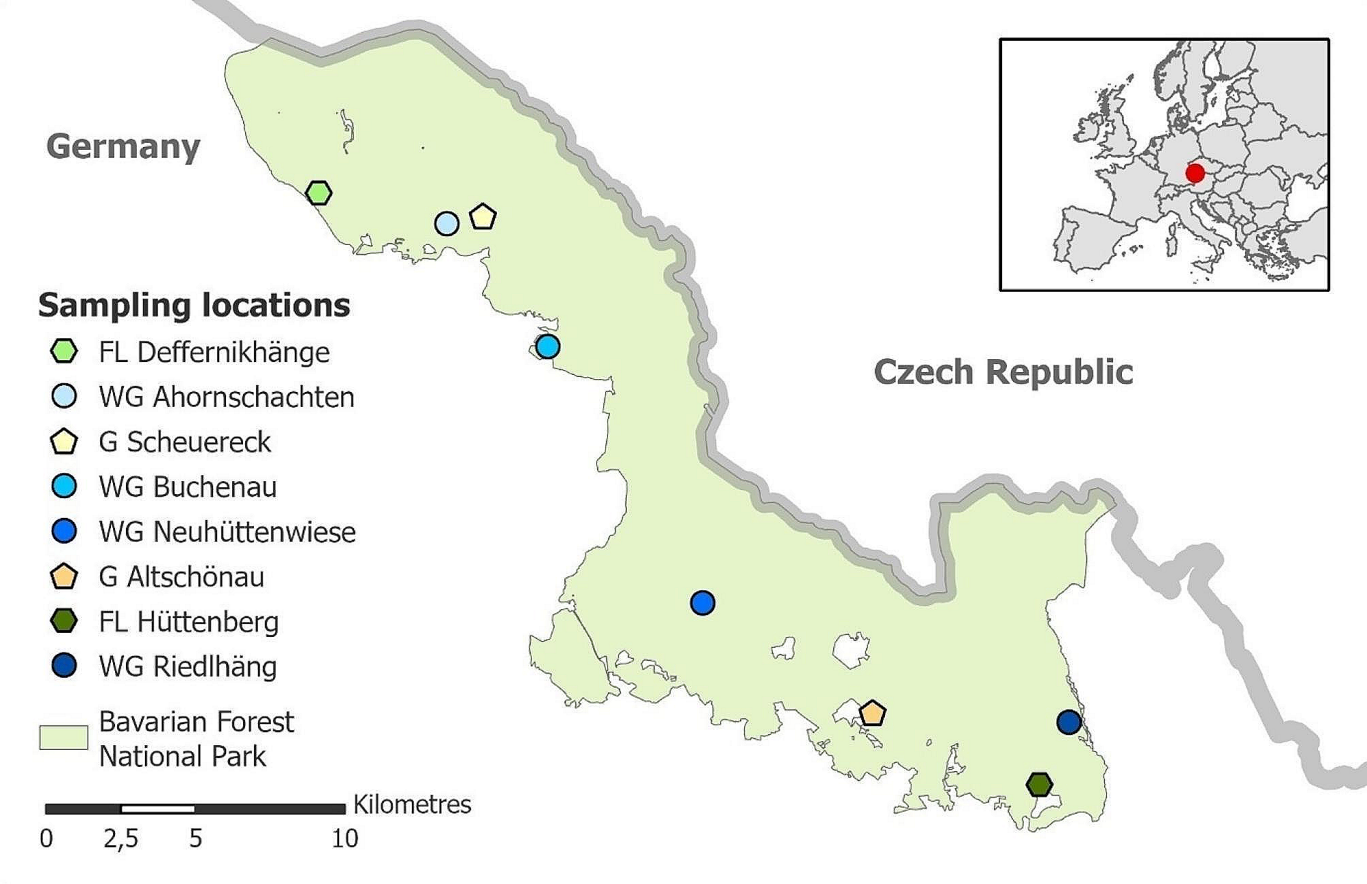
Location of the study area and sampling locations of red deer fecal samples in the Bavarian Forest National Park in southeastern Germany, close to the Czech Republic

The study of the effects of management conditions on red deer gut microbiomes was carried out between 2018 and 2021 in the Bavarian Forest National Park (48°57’13.6 “N 13°23’57.1"E) in southeast Germany (Fig. 1). Along the elevational gradient from 600 m to 1453 m asl, annual mean temperatures decrease from 6.5 °C to 3 °C. Mean annual precipitation ranges between 830 and 2230 mm [29]. We collected fecal pellets from free-ranging individuals (free-living FL: Hüttenberg and Deffernikhänge), semi-captive red deer kept temporarily in four winter enclosures (winter-gated WG: Ahornschachten, Buchenau, Neuhüttenwiese, and Riedlhäng) and two permanently gated enclosures (all-year-gated G: Altschönau and Scheuereck) (Fig. 1).

The winter enclosures for red deer in the Bavarian Forest National Park were established in the 1970s as a wildlife management measure, because the natural winter ranges of the red deer were largely located outside of the designated local red deer territory [30]. In Bavaria, all red deer outside of these red deer territories must be shot by law to prevent damage to agriculture and forestry [31]. Free-living red deer are attracted from October onwards to the winter enclosures by supplementary feeding. The gates are later closed depending on the weather conditions (between October and January). The winter enclosures have an area of 33.04 (± 9.35) hectares with a deer density of 0.41–4.32 per ha. Throughout the winter, the deer diet is supplemented with silage fodder, apple pomace, and sugar beet, which is provided *ad libitum* to the animals [32]. The overall provision of food is regulated quantitatively and qualitatively to fulfill the minimum nutritional requirements of the deer [30], with the daily amount of provided food depending on the number of animals in the enclosure. In spring, between the end of March and the beginning of May when natural vegetation starts to grow again, the gates are opened, allowing individuals to start roaming freely until they return in the following winter [33]. Two permanent enclosures are part of the national park’s educational program, allowing visitors to experience and learn about the animals. The enclosures have an area of 3.8 and 7.8 ha, and the animals are fed with hay, carrots and pellets [34].

We collected 20 fecal pellets, each from one well-defined fecal pellet group (pellets with < 5 cm distance from each other), at the end of winter (April 2018, February 2019, and March 2021) in the permanent enclosures and winter enclosures. Fecal pellets were also collected at locations where free-living deer were present (February 2019 and March 2021). The pellets can be easily distinguished from those of the only other free living ruminant in the study area, the roe deer (*Capreolus capreolus*), by their size and shape [35]. In July 2018, 20 samples were additionally taken at the two permanent enclosures. We only used fresh pellets that were moist and had an intact surface. Like any study gathering samples from wild individuals, there is a risk of sampling the same individual twice: once in the summer (FL) and again in the winter enclosures (WG). While we took precautions to prevent this, we acknowledge that replication is possible. To avoid environmental contamination, we dissected the fecal pellets in the field by using sterile equipment, collecting only the inner part of the pellet and preserving it in 1.5 mL Eppendorf tubes containing 600 µL nucleic acid preservation buffer (own formulation) [32, 36]. All samples were stored at -20 °C until DNA extraction.

### DNA extraction

We extracted total DNA from the fecal pellets by using the NucleoSpin Soil Kit 96 (Macherey-Nagel, Düren, Germany). We followed the manufacturer’s guidelines with two minor modifications to optimize the protocol. We used about 150 mg wet fecal matter as the starting point of the extraction and performed two consecutive DNA elution steps at the end of the protocol to ensure a total yield volume of 100 µl. We also extracted several negative controls to account for contamination during the extraction process. The extracted DNA was stored at -20 °C in 96-well plates.

### Amplification of the 16 S rRNA gene V4 region, library preparation, and Illumina sequencing

We targeted a 291-bp fragment of the hypervariable V4 region of the 16 S rRNA gene with the universal bacterial primers 515 F (′5-GTGCCAGCMGCCGCGGTAA-3′) and 806R (5′-GGACTACHVGGGTWTCTAAT-3′) following the earth microbiome protocol and recommendations for polymerase chain reaction (PCR) amplification [37]. We used a SimpliAmp Thermal Cycler (Applied Biosystems, Darmstadt, Germany) for the amplification with a two-step PCR approach. In the first PCR, the target was amplified using the primers 515 F and 806R, and during the second PCR, individual barcodes and the Illumina adapters were added. The primers were tagged with universal adapters (CS1 and CS2, Standard BioTools, South San Francisco, USA) and 4 Ns were added to the forward primer for cluster identification during sequencing. A prepared target-specific primer mix (TS) contained CS1-4 N-515 F and CS2-806R, 400 nM each. The first PCR included 1 µl template DNA, 5 µl AmpliTaq Gold™ 360 Master Mix (Applied Biosystems, Darmstadt, Germany), 1.5 µl TS primers, and 2.5 µl water to give a final volume of 10 µl. Our amplification protocol included an initial warm-up at 95 °C for 10 min, followed by 30 cycles with 30s at 95 °C (denaturalization), 30s at 60 °C (annealing), and 45s at 72 °C (elongation), with a final elongation at 72 °C for 10 min. For the 20 µl barcoding step, we used 10 µl AmpliTaq Gold™ 360 Hot Start Master Mix (Applied Biosystems, Darmstadt, Germany), 3 µl template amplicon from the unbarcoded sample, 4 µl individual barcodes and Illumina adapters (Fluidigm Access Array™ System for Illumina Sequencing Systems, ©Standard BioTools, South San Francisco, USA), and 3.0 µl ultrapure water. The reaction times were identical to those in step one, but only 10 cycles were carried out.

We purified and cleaned up residual oligonucleotides by using NucleoMag® NGS Clean-up and Size Select Kit (Macherey-Nagel, Düren, Germany) on a GeneTheatre® (Analytik Jena, Jena, Germany) according to the manufacturer’s guidelines and our own workflow stream and checked for the expected amplicon length by using capillary electrophoresis on a QIAxcel Advanced System (QIAGEN, Hilden, Germany).

We measured DNA concentrations of the barcoded samples by using the fluorescent dye of the QuantiFluor® dsDNA System (Promega, Madison, USA) on a TECAN Infinite F200 PRO® plate reader (Tecan, Männedorf, Switzerland). We normalized the samples to include 60 ng of each indexed amplicon in the final library. For sequencing, we used an 8 pM library loaded onto a MiSeq flow cell and spiked our library with *PhiX* sequencing control V3 at 5% (Illumina MiSeq Reagent Kit V2). Paired-end sequencing was performed over 2 × 251 cycles in an Illumina MiSeq (Illumina, San Diego, USA) following the manufacturer’s recommendations.

### Bioinformatic processing

We used the QIIME2 Command Line pipeline (version 2020.2) in a Linux Mint 19.2 environment for demultiplexing and denoising. After removing the adapters and primers, we consolidated our sequence library by keeping 200 base pairs in each reading direction. The mean quality score for this part of the sequence was 37. Both the sequence length and the quality score were selected to maximize sample retention without decreasing the sample quality and following the standard established by other papers [32, 38, 39]. We chose the DADA2 plugin [40] to account for sequencing error rates, to form consensus sequences, and to remove chimeras and other artifacts [40]. We assigned taxonomy to each Amplicon Sequence Variant (ASV) by training a SILVA V4 Classifier (SSU release 138 515–806) object with the “*qiime feature-classifier classify-sklearn*” function in QIIME2 at the highest level of taxonomical resolution (level 7). We purged our dataset of any sequences assigned to chloroplasts, mitochondria, and archaea or of that given the “Unassigned” tag. The curated data base was imported into R [41] and the *phyloseq* package was used for all subsequent analysis [42]. We then removed the taxa that were identified in the extraction and PCR controls as contaminants and kept only samples that retained at least 8,000 reads following the filtering steps in our database.

### Statistical analysis

To construct compositional plots, we consolidated our database down to a higher taxonomical resolution (family and phylum level) and calculated the relative abundance of the specific taxa in each sample according to the sample ID and management condition (FL, WG, G). To simplify the visualization, we sorted all taxa with a relative abundance lower than 3% into a new category named “Others”.

To assess individual microbial alpha diversity, we used the number of Amplicon Sequence Variants (ASVs) to represent the taxa richness [43], the Shannon-Wiener index [44, 45] as a proxy for the community entropy, and lastly, Faith’s Phylogenetic diversity (Faith’s PD) index [46] to consider phylogenetic diversity. We calculated an index for each sample and then collated the resulting information by sampling locality and management conditions (FL, WG, G). Because of the non-normal nature of the data, we used the Wilcoxon test [47] to ascertain overall differences between groups and the Kruskal-Wallis test [48] for pairwise differences between management conditions. We used the False Discovery Rate correction (FDR) to correct all P-values when multiple tests were performed.

Before beta diversity calculations, we filtered out all singletons from the data and applied a prevalence filter to our data to remove ASVs that had not been present in at least 40% of the samples. Finally, we removed any sample remaining that had fewer than 12,000 reads from the database. This step is necessary since by applying a 40% of samples threshold, some samples are left with just a few ASVs that account for a small number of reads. By applying the filter, we are ensuring a sufficient depth of coverage for the samples that are included in the analysis.

We used a dissimilarity matrix to calculate the two beta diversity metrics, namely Weighted and Unweighted UniFrac distances between individuals [49–51]. The Weighted UniFrac distance metric integrates the taxonomic richness and the relative abundance of a specific ASV; therefore, it shows the difference between the most abundant features of the individual bacterial microbiota. The Unweighted UniFrac disregards the relative abundance of any particular ASV; hence, it is mainly influenced by presence-absence data. We used the first two variables as ordination axes and plotted the data according to management condition and/or sampling locality to reduce the number of dimensions and to provide a visual approximation to the difference between management conditions. We employed a Permutational Multivariate Analysis Of Variance Using Distance Matrices [PERMANOVA [52]], to test for differences in beta diversity measurements between groups and evaluated the statistical significance using the Adonis function in the “*vegan*” package by using 9,999 permutations [53].

To infer bacterial microbiota heterogeneity, we calculated the centroids of all pairwise distances for each group and plotted the centroids together with all data points and their 95% confidence ellipses. We used the data dispersion around the centroid for each management condition to represent variability within the groups. We tested for differences between dispersion by means of the “*betadisper*” function in the *Vegan* package in R [53].

Finally, to test for the differential abundance of specific ASVs, we employed an analysis of compositions of bacterial microbiotas, namely ANCOM v.2 [54]. This method allowed us to distinguish between sampling and structural zeroes in the data (see [55] for details). We created a volcano plot mapping by family and genus for the differentially abundant taxa.

## Results

We collected 485 samples distributed across the three different management conditions (FL, WG, G) and years (Suppl. Table 1). After filtering out the controls and removing all contaminants, our final database contained 13,648,922 reads encoding 27,942 unique Amplicon Sequent Variants (ASVs) distributed across all samples. Following the removal of all reads from any sample with less than 8,000 reads, each sample had on average 27,667 (± 7,322) reads and an average of 465 (± 134) unique ASVs.

### Effect of management on gut bacterial community composition of red deer

**Fig. 2:**
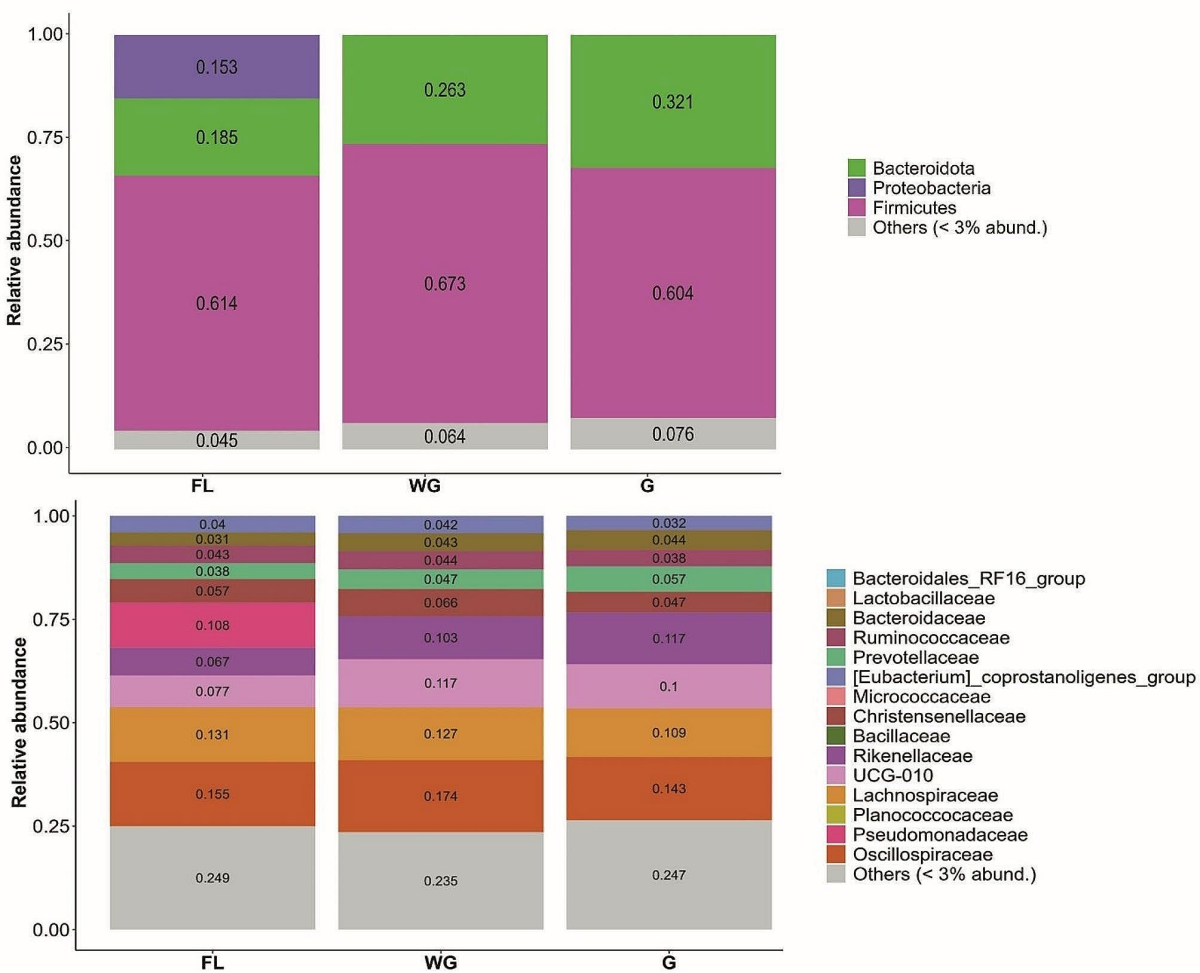
Mean bacterial composition of the gut bacterial microbiotas of red deer living under three management conditions (free living (FL), winter-gated (WG), and all-year-gated (G)) in the Bavarian Forest National Park at phylum (top panel) and family (bottom panel) level

The bacterial microbiota composition was more similar in animals experiencing semi-captive and captive management conditions, compared with free-living red deer (Fig. 2). The bacterial microbiota of WG and G-individuals were mainly composed of Firmicutes (60.1–67.1%), followed by Bacteroidota (24.6–31.2%). The free-living individuals also had bacterial microbiota firmly founded on Firmicutes (61.4%) and Bacteroidota (18.5%) but additionally harbored a substantial contribution from Proteobacteria (15.3%). At the family level, the composition was stable across the different management conditions, but the free-living individuals had a substantially higher representation of the Pseudomonadaceae family (10.8%).

### Effect of management on gut bacterial alpha and beta diversity pattern and heterogeneity of red deer bacterial microbiotas

**Fig. 3:**
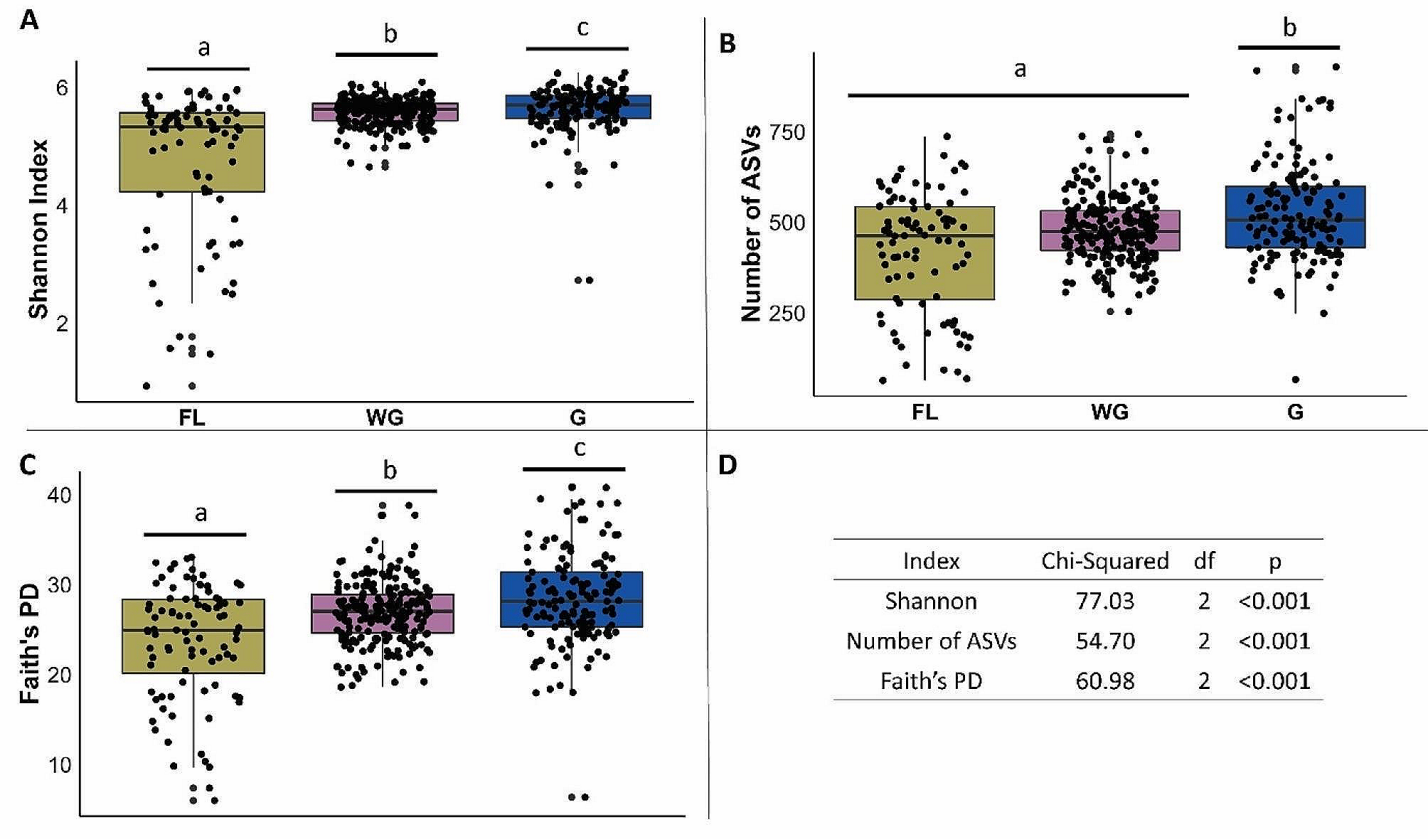
Differences in alpha diversity metrics of the gut bacterial microbiotas of red deer living under three management conditions (free living (FL), winter-gated (WG), and all-year-gated (G)) in the Bavarian Forest National Park. (**A**) Shannon diversity index, (**B**) Number of amplicon sequence variants (ASVs), and (**C**) Faith’s Phylogenetic Diversity. (**D**) shows results from the the Kruskal-Wallis test for each alpha diversity metric

The alpha diversity indices, entropy (Shannon-Weiner) (Fig. 3A), bacterial taxa richness (number of ASVs) (Fig. 3B), and phylogenetic diversity of the individual bacterial community (Faith’s PD) (Fig. 3C) increased with the level of management and confinement. Accordingly, we found that all-year-gated animals had a significantly higher taxa richness (number of ASVs) than free-living deer and individuals under semi-captive conditions (Fig. 3). The free-living individuals (FL) had the lowest scores for these indexes (Fig. 3B-C). Altogether, the trend showed that animals under more constrained management conditions had higher alpha diversity scores than free-living red deer.

**Fig. 4:**
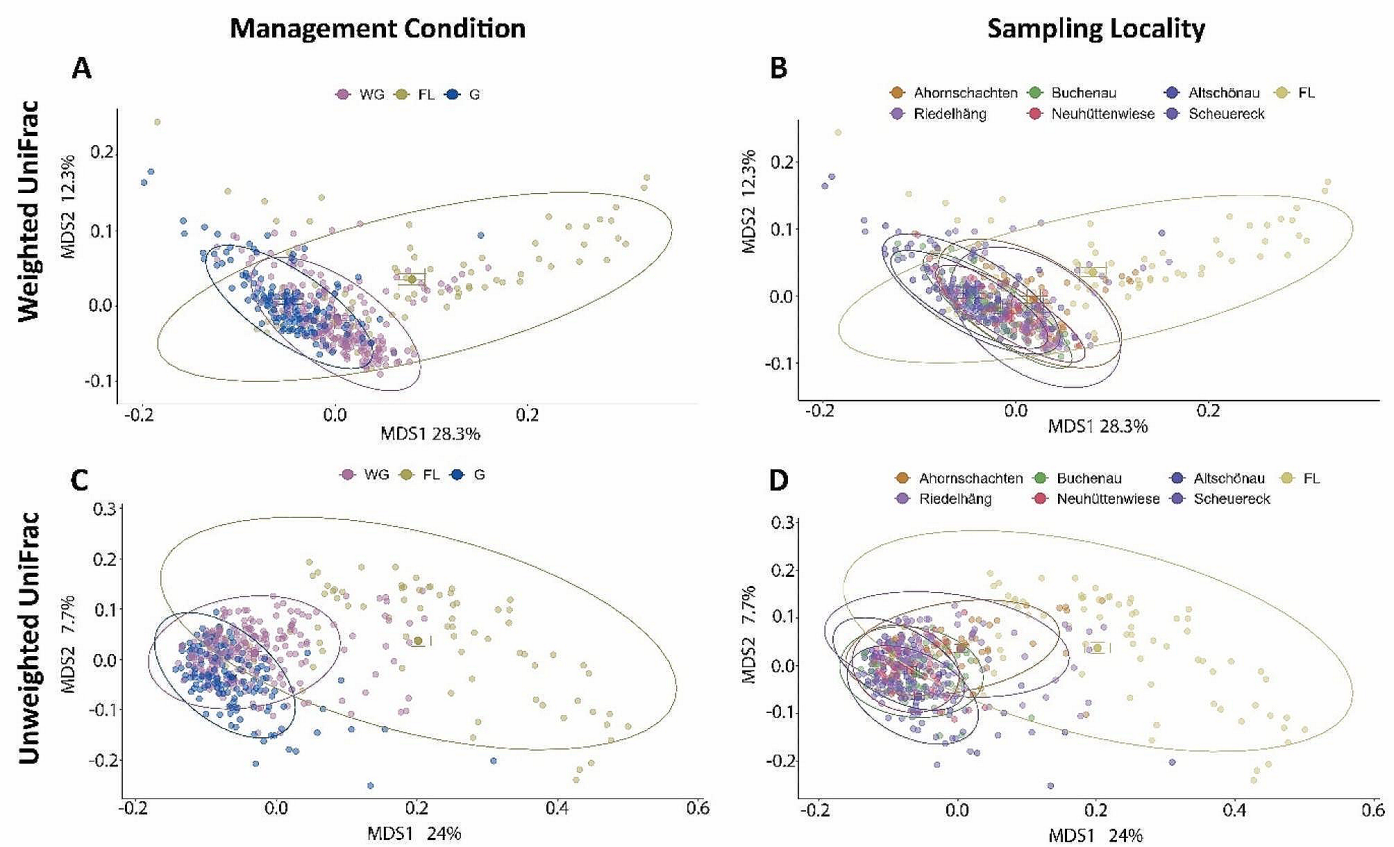
Differences in beta diversity metrics of the gut bacterial microbiotas of red deer living under three management conditions (free living (FL), winter-gated (WG), and all-year-gated (G)) in the Bavarian Forest National Park. (**A**-**B**) Weighted and (**C**-**D**) Unweighted UniFrac distances for each individual by management condition (**A**, **C**) and by sampling locality (**B**, **D**)

Regarding beta diversity, samples clustered according to management conditions and sampling locality (Fig. 4). Both management condition and year-to-year variation were determining factors for beta diversity in the Weighted and Unweighted UniFrac metrics (Suppl. Table 2). Overall, we obtained a clear result: the free-living individuals had a lower alpha diversity at the individual level, but a more diverse and heterogenic bacterial microbiota was observed among these individuals than among the individuals under the other two management conditions (WG, G).

**Fig. 5:**
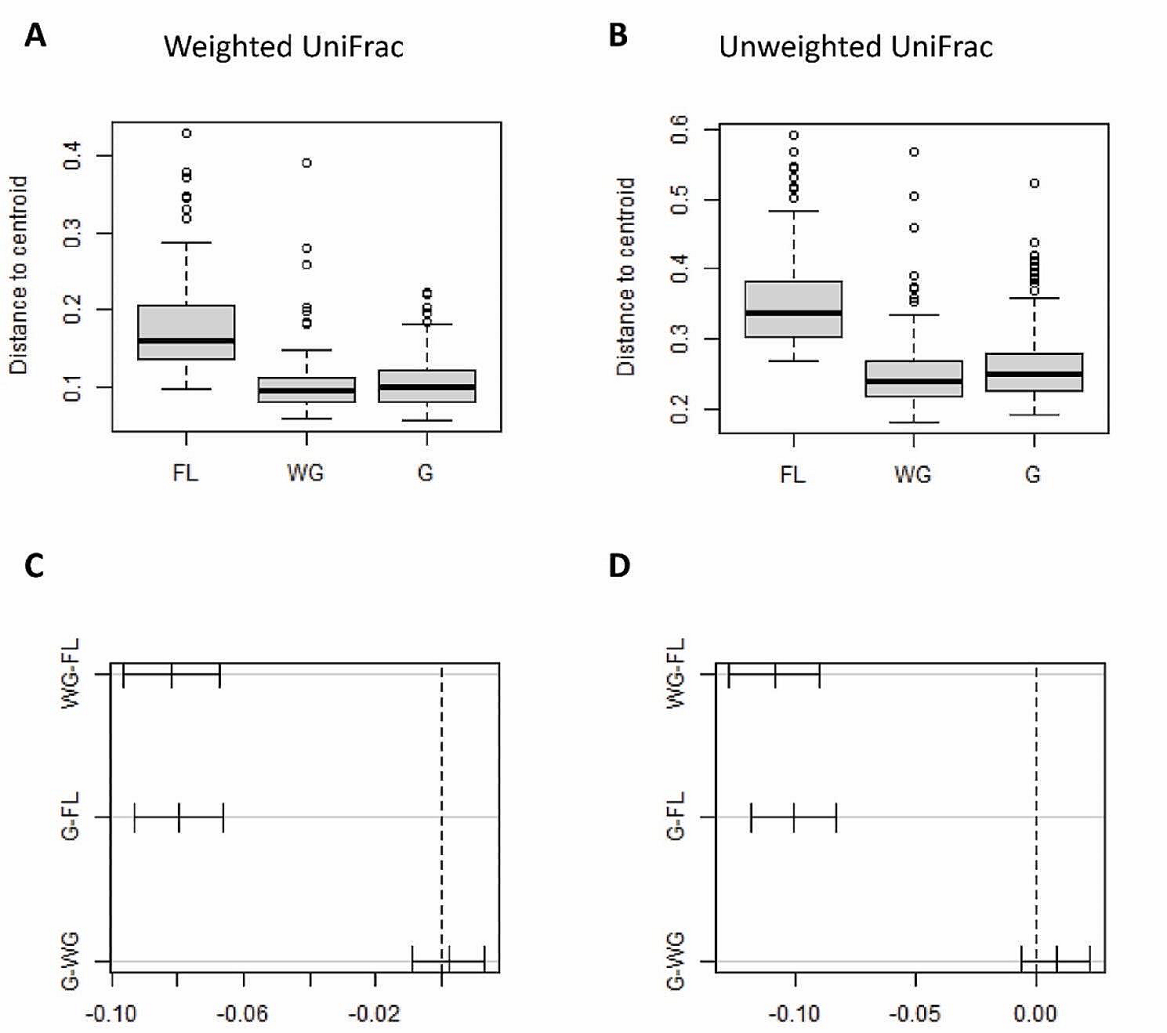
Data dispersion (bacterial microbiota heterogeniety, i.e., relative distance of each individual to the group centroid) within each management category (FL, WG, G) for both (**A**) Weighted and (**B**) Unweighted UniFrac distances. (**C**-**D**) show the results of pairwise comparisons between management conditions

Bacterial microbiota heterogeneity, meaning the distance of a particular individual to the centroid of its group, decreased with more restrictive management conditions. This effect was consistent for both core (Weighted UniFrac distances) and non-core (Unweighted UniFrac distances) bacterial microbiota features. Thus, we detected higher levels of variation in free-living individuals not just for the rare taxa, but also for the most abundant and resilient components of the bacterial microbiota of the red deer at our study sites. Moreover, the data dispersion was significantly higher in the FL individuals than in WG and G individuals (Fig. 5).

### Effect of management on differential abundance of bacterial taxa

**Fig. 6:**
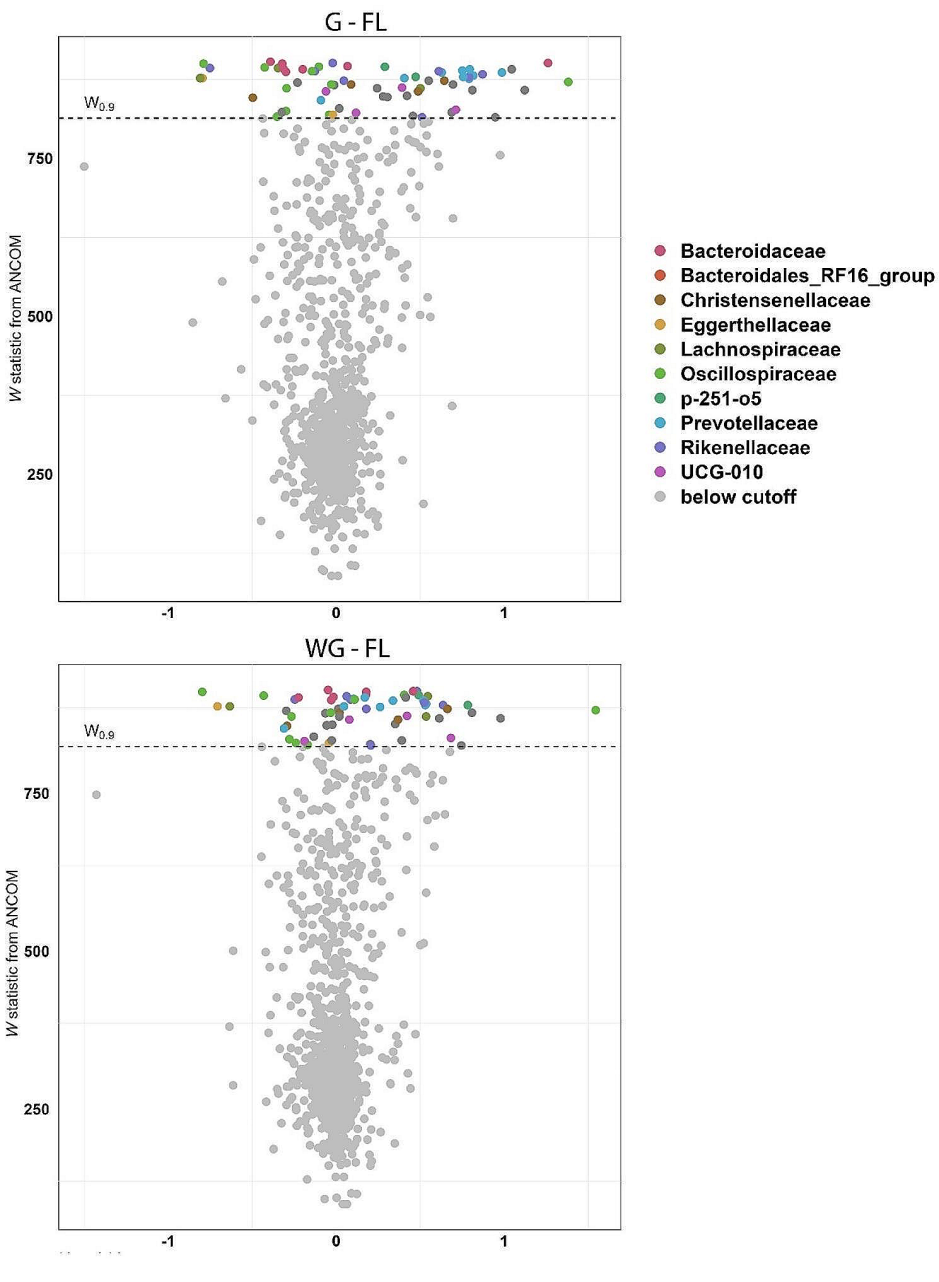
Effect of red deer management on differential abundance of bacterial taxa illustrated by volcano plots. Top panel: comparison between free living (FL) and all-year-gated individuals (G); bottom panel: comparison between between free living (FL) and winter-gated individuals (WG)

Several bacterial taxa differed in abundance between the management conditions based on ANCOM. The gut bacterial microbiota of the free-living red deer harbored several exclusive taxa, such as *Treponema* (Treponemataceae), *Ruminococcus* (Oscillospiraceae), *and Bacteriodes* (Bacteroidaceae). We also found that bacterial taxa such as *Roseburia* and *Parvibacter* were more abundant in the free-roaming individuals than in deer held under the other two management conditions. Other bacterial families such as Lachnospiraceae, Rikenelleaceae, Flavobacteriaceae, and Eggertheliaceae were also significantly more abundant in free-living individuals (Fig. 6).

On the contrary, some Bacteroidaceae, including members of P-251-05, were more abundant in winter-gated and gated individuals. The gated individuals also presented a high abundance of Prevotellaceae (UGC-004), a well-known endosymbiont of ruminants, and a higher abundance of a different taxa of *Bacteriodes*.

## Discussion

Animals act as host to complex inner ecosystems consisting of a plethora of microorganisms that not only inhabit and metabolize food, but actively contribute to the overall immune condition of individuals [56, 57]. Environmental and associated dietary shifts might impact gut microbial communities. In extreme cases, changes in diet regimes drive (A) the loss of homeostasis typically exemplified by a decline of commensal bacteria and (B) an increase of pathogenic taxa. In the present study, we tested the impact of food provisioning, which forms part of the wildlife management concept of the Bavarian Forest National Park (Germany) during the winter, on the gut bacterial microbiota composition of red deer. Moreover, we compared the gut bacterial microbiota of all-year free-ranging individuals with those of individuals spending the wintertime in large enclosures and with those of individuals that are all-year-gated for tourism purposes.

Regardless of the management condition all individuals harbored a typical, healthy artiodactyl gut bacterial microbiota composition [58, 59]. Following our expectations, the bacterial microbiota was more similar between the groups of animals that spent at least part of the year in captivity. Although differences were notable in terms of composition, all treatment groups followed a well-structured and predictable composition for an herbivorous artiodactyl bacterial microbiota strongly dominated by Firmicutes and Bacteroidota [32, 60, 61]. Firmicutes dominate nutrient absorption and bioavailability because of their high capacity for hydrolyzing carbohydrates [62, 63]. Additionally, we found that the bacterial microbiota composition of the free-living deer had a higher contribution of Proteobacteria.

### Captive red deer had a higher gut bacterial microbiota alpha diversity than their wild conspecifics

Interestingly, we observed that captive red deer in the Bavarian Forest National Park had a higher gut microbiome alpha diversity than their wild conspecifics, whereas the individuals that overwintered in enclosures were ranked in between. The alpha diversity was higher in gated animals for all metrics (ASVs, Shannon, and Faith’s PD) compared with the other treatments. During the winter, free-ranging individuals feed on grasses and browse trees and shrubs [64]. Also, individuals in the large temporary gated winter enclosures can feed on natural vegetation but additionally obtain silage, mashed apples and sugar beets, whereas all-year-gated red deer solely rely on artificial food provisions. Similar to our observations for red deer in the Bavarian Forest, sika deer (*Cervus nippon hortulorum)* kept in gated enclosures on farms have a higher alpha diversity than free-living sika deer [65].

McKenzie et al. [66] showed that the microbiome response to captivity is extremely taxa-dependent, and that, for some species, captivity had no effect on alpha diversity (bovids, giraffes, anteaters, and aardvarks), whereas it had a negative effect in other taxa (canids, primates, and equids) and a positive effect in two species (Black and White Rhinoceros). In our study, we found similarly, that red deer in yearlong captivity had higher alpha diversity of bacterial gut microbiota than the semi-captive and free ranging individuals. McKenzie et al. did not discuss potential explanations for the increased alpha diversity in captive rhinoceroses. Presently, we can also not explain the increase of alpha diversity in captivity that we observed for red deer. In the future, this relationship could be explored using an experimental approach. For ruminants in general, McKenzie et al. propose that a stable gut microbiome is able to utilize a variety of feeds and does not result in differences between free-living and captive animals [66, 67]. In agreement with this notion, the microbiome alpha diversity of roe deer (*Capreolus capreolus*) receiving supplemental feeding, of captive forest musk deer (*Moschus berezovskii*), and of captive Père Davids deer (*Elaphurus davidianus*) is marginally lower but not significantly different compared to that of their conspecifics living on a natural diet in the wild [59, 61, 68].

### Free-living individuals had a higher beta diversity and hosted a more heterogeneous gut microbiome community than their captive counterparts

Contrary to the alpha diversity patterns, the beta diversity between individuals who lived either temporarily or year-round in enclosures was strongly reduced. Beta diversity showed a nested pattern in which individuals from the same management category had more similar microbiomes, i.e., captive and semi-captive red deer had more similar gut microbiomes than free-living individuals. This trend was sustained both for the core (Weighted UniFrac) and non-core (Unweighted UniFrac) features of the microbiome. We observed that the centroids of both the captive and semi-captive individuals were closer to each other than to those of the free-living individuals. Moreover, free-living individuals host a more heterogeneous gut microbiome community than their captive counterparts, meaning that beta diversity increases with a natural diet.

Following the assumption that the microbiome is governed by a combination of diet, environment, and phylogeny [69, 70], it is not outrageous to attribute the heterogeneity of the environment that free-living animals face on a day to day basis (and the inherent expanded dietary offer) as a determining factor in microbiome composition and heterogeneity between individuals. Free-ranging animals are exposed to much more divergent conditions than their captive or semi-captive counterparts. Red deer are classified as intermediate feeders, foraging on a wide variety of plant parts from different species, including graminoids, forbs, and fruits, plus leaves, needles, and the bark of bushes and trees [71]. In the Bavarian Forest National Park, red deer primarily feed on grasses and deciduous and coniferous trees and browse on bilberries, ferns, and bramble during the fruiting season, dependent on the habitat. The proportion of parts of coniferous trees in the diet increases strongly in winter [64]. The food items that the deer select from this diverse palette can differ strongly between individuals, and these differences can, for example, be shaped by the foraging patterns of their mothers [72].

Interestingly, the animals in the winter enclosures showed a similar pattern to the all-year-gated individuals, although they are free-ranging for most of the year and have no direct contact with the gated animals. In addition, the enclosures are not clustered, but distributed across the landscape, evidencing a negligible spatial component in the microbiome adaptation (Fig. 1). The similarity must therefore be connected to the similar composition of their artificial diet, with a large proportion of silage and other agricultural items. A consistent change of diet for less than a week can alter the microbiome composition and beta diversity drastically [73]. Overall, diet is a significant force that shapes the microbiome [69, 73–75], although the higher contact rate between individuals [76] in the winter enclosures and permanent enclosures might also contribute to the homogenization of the microbiome compositions between the animals.

This raises the question of why microbiome heterogeneity is critical for the survival of wildlife populations. The microbiome makes substantial metabolic and physiological contributions to the host’s health, being referred to as the third genome of a host (with the first two being nuclear and mitochondrial). As for all other genetic components, a divergence between individuals might be critical, as selection forces associated with environmental and associated dietary changes inflict differential pressures on different genomes. Microbiome heterogeneity mimics bacterial genetic diversity (homologous to the fixation index (F_ST_) in population genetics), which is crucial for the survival and resilience of healthy animal populations.

The timing of sample collection and winter confinement likely contributed to the striking similarity between the gut microbiota of WG and G animals. Research has shown that seasonal changes can significantly impact the composition of gut microbiota in White-lipped deer (*Cervus albirostris*) [77], and environmental factors such as diet and housing conditions can also influence gut microbiota. Therefore, it is probable that the prolonged winter confinement and sampling time played a vital role in shaping the gut microbiota composition of the WG and G groups in our study. These findings emphasize the importance of considering the timing of sample collection and environmental factors when studying the gut microbiota of animals.

### Free-ranging individuals harbored a higher abundance of specific bacterial taxa attributed to host health

An exclusive bacterium in free-ranging individuals was *Ruminococcus* sp. This genus is a common gut symbiont of Cervidae and has been reported in wild sika deer in China [62] and captive elk in South Korea [58] An experimental study on Norwegian reindeer (*Rangifer tarandus*) has shown that, when animals are dosed with probiotics (including *Ruminococcus flavefaciens)*, the overall microbiome diversity (Faith’s PD) and evenness decreases significantly [78]; demonstrating that this bacterium can significantly shape the diversity and structure of the gut microbiome. In mice, *Ruminococcus gnavus* is an essential link in the brain-gut axis, and animals treated with this bacterium showed improvements in the modulation of granule cell development and spatial memory enhancements [79]. Both these studies highlight the importance of keystone bacteria in the gut and present the question as to how a specific bacterium can shape the gut microbiome structure and diversity and the brain-gut axis.

The free-living deer in our study also had a higher abundance of *Roseburia* than their captive and semi-captive counterparts. This bacterium (especially *Roseburia intestinalis*) has been highlighted as a good health marker. *Roseburia* is an anaerobic gram-positive bacterium that produces high levels of short-chain fatty acids (SCFAs) in the colon [80]. These SCFAs play not only a significant role in nutrient bioavailability and T-cell generation [81, 82], preventing inflammation [83, 84], but also form an essential link in the brain-gut axis [85].

However, we have not only detected beneficial bacteria in free-ranging individuals. Their gut microbiome also harbors *Treponema* (Treponemataceae). *Treponema* is a large genus of bacteria containing human and wildlife pathogens [86]. In humans, *Treponema pallidum pallidum* is the causative agent of syphilis [87]. In artiodactyls, *Treponema* has been found to be the pathogen responsible for digital dermatitis in elk in North America [86, 88]. A recent study has found *Treponema* in fecal samples of free-ranging red deer from Portugal [89]. These samples also present high levels of Tetracycline antibiotic resistance genes (ARGs). However, the individual contribution of this bacterium to microbiome-wide signals is difficult to distinguish.

## Conclusions

We have found that the supplemental feeding of red deer is correlated with higher alpha diversity of the bacterial gut microbiota, emphasizing the role that supplemental feeding plays in shaping the gut microbiome of individuals. Our study demonstrates that even temporary dietary changes have an impact on the gut microbiome and, thus, the importance of properly monitoring gut health when animals are translocated or kept in temporary enclosures (i.e., animals in quarantine).

Additionally, our study shows that gut microbiome heterogeneity decreases with the degree of supplemental feeding. Animals that feed freely have gut compositions that are more different from each other when compared with animals that receive supplemental feeding. Such microbiome homogenization might be explained by less diverse foraging options and increased contact between individuals when held in gated or temporarily gated conditions. This information is crucial for wildlife management, since microbiome homogenization can lead to faster pathogen transmission within a population. Our study highlights the value of implementing microbiota investigations as an important health marker in wildlife management and conservation.

### Electronic supplementary material

Below is the link to the electronic supplementary material.

Supplementary Material 1

## Data Availability

Upon acceptance, data will be available for the public by request to the authors.
