# Supplementary material for: A taste of wilderness: supplementary feeding of red deer (Cervus elaphus) increases individual bacterial microbiota diversity but lowers abundance of important gut symbionts
Source: Anim Microbiome. 2024 May 14;6:28. doi: 10.1186/s42523-024-00315-6 (PMC11094858; doi:10.1186/s42523-024-00315-6)

Supplementary Table 1. Red Deer faecal sample collection by management condition and year in the Bavarian Forest National Park, Bavaria, Germany.

| Condition/Year | **2018** | **2019** | **2020** | **2021** |
| --- | --- | --- | --- | --- |
| **FL** |  | 40 | 19 | 29 |
| **WG** | 76 | 87 |  | 85 |
| **G** | 72 | 38 |  | 39 |
|  |  |  |  |  |

Supplementary Table 2. Coefficients and results of the PERMANOVA analysis for both the Unweighted and Weighted UniFrac.


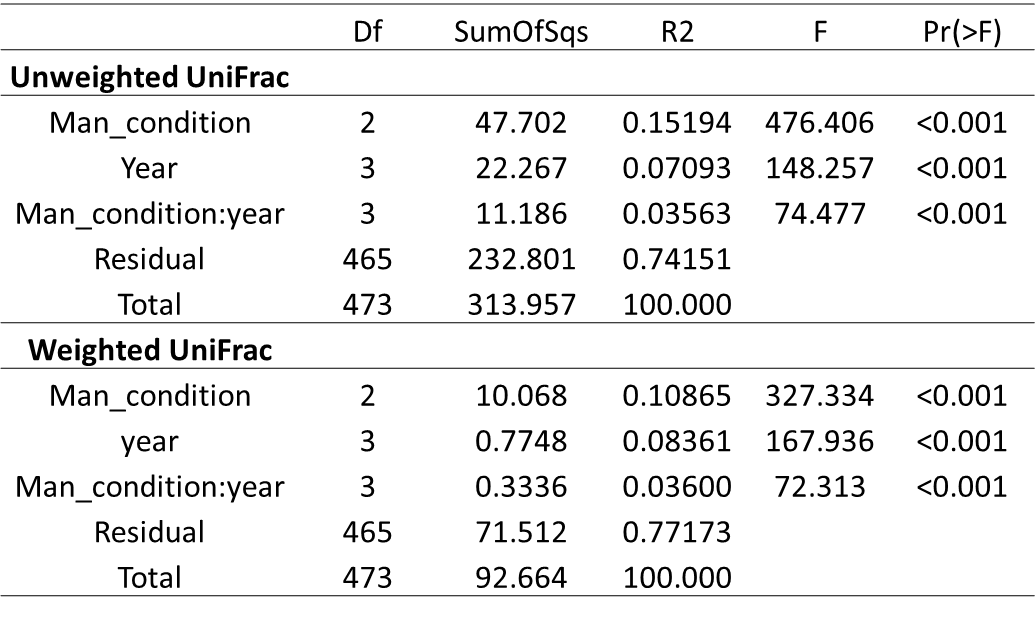

Supplement: Supplementary file 1 — Supplementary Material 1 [file 42523_2024_315_MOESM1_ESM.docx]
